# The “Land of Fires” Toxic Waste Scandal and Its Effect on Consumer Food Choices

**DOI:** 10.3390/ijerph16010165

**Published:** 2019-01-08

**Authors:** Luigi Cembalo, Daniela Caso, Valentina Carfora, Francesco Caracciolo, Alessia Lombardi, Gianni Cicia

**Affiliations:** 1 Department of Agricultural Sciences, University of Naples Federico II, 80138 Napoli NA, Italy; cembalo@unina.it (L.C.); francesco.caracciolo@unina.it (F.C.); alessialom@gmail.com (A.L.); cicia@unina.it (G.C.); 2 Department of Humanities, University of Naples Federico II, 80133 Napoli NA, Italy; 3 Department of Psychology, Catholic University of Milan, 20123 Milano MI, Italy; valentina.carfora@unicatt.it

**Keywords:** environmental scandal, regional food safety, theory of planned behavior, Land of fires, Campania, Italy

## Abstract

The present study focused on an environmental scandal that occurred in Italy, the *Land of Fires* toxic waste scandal, which caused consumer concerns related to the safety of food produced in the affected region, as well as massive market reduction in products associated with the polluted area. Based on a representative sample of Italian households (*N* = 1134), this study applied an extended Theory of Planned Behavior (TPB) model to analyze consumer purchases of regional food products after this environmental hazard. In addition to attitudes, subjective norms and perceived behavioral control, the model included risk perception, trust, and actual purchases. Using a structural equation model, our results provided support to the hypothesis that consumer perceptions of risk negatively impacted their purchase behaviors and suggested that increasing Italians’ trust in government information could reduce their perceived risk and, consequently, increase their intention to purchase regional food.

## 1. Introduction

Over recent decades, many environmental psychologists have analyzed the perceptions of environmental hazards and beliefs surrounding how these hazards should be managed. For instance, many authors have analyzed the level of concern over environmental hazards such as climate change [1] or air pollution [2]. In the public media, increasing attention has recently been focused on the environmental damage connected to food scandals. Due to an increase in the frequency and severity of environmental damage, food sectors have been routinely exposed to the risk of growing food insecurity. Several safety scandals, in turn, have caused substantial economic shocks with related shifts in the consumption patterns, either directly or indirectly, of affected food products [3]. Thus, environmental scandals are raising a more general issue of food safety that is globally considered a prerequisite for both public health and market development [4,5].

Food safety incidents are not new phenomenon, and the list is wide, including wine adulteration with methanol, bovine spongiform encephalopathy, and bird flu [6,7,8,9]. Food safety incidents can be caused by potential undesirable residues in foods derived from environmental incidents, for example, dioxin in cheese, sulfur chemicals or atrazine in drinking water, and heavy metals in food crops [10,11,12,13]. The effects of environmental contamination on food safety have severely undermined the confidence of consumers in the food industry [14]. In fact, consumer concerns surrounding food are connected to health as well as agriculture, ecology, and food culture. Environmental changes associated with food production, such as contaminated farmland and the use of pesticides, appear to be vitally important to society and are of increasing interest to consumers [15]. Scholars have focused much of their attention on this topic. Economic effects in the short and long-term along food supply chains that result from the psychosocial response of consumers in the market has been investigated in the context of several food safety scandals [16,17,18,19,20]. Other contributions in the literature studied not only how to minimize the likelihood that similar events occur again but also the role of information in creating fear as well as the role of institutional reassurance strategies [3,21,22,23]. To the best of our knowledge, within the environmental psychology domain, no scholars have focused on the modern environmental hazards that lack food safeguards. These hazards include water pollution, urban air pollution from automobiles, solid and hazardous waste accumulation, toxic waste dumping, chemical and radiation hazards, and heavy metals in farmland soils [24]. Such environmental hazards often produce drastic consequences in terms of individuals’ food purchasing behaviors.

The current study contributes to this debate by investigating the beliefs of Italians regarding the recent discovery of an environmental hazard with food scandal effects. The present study investigated how Italian consumer cognitions, such as attitudes, risk, and trust, affect their food purchases after an environmental disaster, namely, the *Land of Fires* toxic waste scandal. This scandal occurred in Italy in 2013–2014 in an area located on the northern-east side of the Campania region (Southern Italy), once known as “*Campania felix*” (lucky Campania) because it was one of the most fertile lands in the entire Roman empire [25]. After this environmental scandal, this area was renamed *Land of Fires* by one of the best-known Italian environmental organizations (Legambiente: League for the Environment) because of the widespread practice of illegally dumping and burning toxic waste of various types, including chemicals, heavy metals, petroleum and more by organized crime members. *Land of Fires* is also a territory where a high cancer-related mortality was observed [26]. This area is known for producing popular traditional food products, all benefiting from protected designation of origin (PDO) status, such as water-buffalo *mozzarella* cheese, water-buffalo *ricotta* cheese, canned *San Marzano* tomatoes, *Piennolo* tomatoes, *Falerno*, *Asprinio* and *Falanghina dei Campi Flegrei* wines. The national reputation of that area has, in recent years, not been positive, but it never affected the demand of the abovementioned products before 2013. In 2013 and 2014, the consumer demand for these products experienced a dramatic collapse. As an example, the water-buffalo *mozzarella* cheese sector suffered a decrease in revenues by approximately 57 million Euros in 2014 [27]. An analogous trend was also encountered for the other abovementioned products, which are major contributors to the regional added value. It is of common opinion that such a dramatic market decline was due to the *Land of Fires* toxic waste scandal. The *Land of Fires* toxic waste scandal was characterized by a consequent food scandal, since the media attempted to link environmental crimes occurring in the *Land of Fires* to food production in the same area. Although this link between the environmental crimes and the food security was never scientifically assessed, some media have generated doubt in the safety of food produced in this area. Consequently, a massive market reduction in these products occurred almost immediately. National and regional institutions made a huge effort to reassure consumers on the safety of the food produced in that area. Scientific information was widely provided to consumers. However, the reluctance of consumers to purchase Campania food products has remained.

The above considerations give rise to the hypothesis that consumers, when facing news of an environmental scandal, react based on their own judgment about the potential risk of consuming food produced in the polluted area and about the trust they have in public institutions. The innovative contribution of this paper is to consider the impact of *Land of Fires* toxic waste scandal on Italian consumers by using the psychological domain as its theoretical framework. The current research adopted an environmental psychology perspective, considering that the perceptions of consumers surrounding an environmental hazard can strongly affect the purchasing of authentic regional food produced in the polluted area, even when the food environmental hazard was not scientifically associated with the safety of this food. If we want to devise policies and actions that aim to deal with the environmental scandals and their related crisis in food purchases, we need to have a more sophisticated theoretical understanding of consumer cognitions and their consequent purchase behaviors. Environmental psychological insights surrounding consumers’ reactions to this environmental crime may aid in policymaking to resolve this environmental, social and food problem.

### 1.1. Research Framework

The Theory of Planned Behavior (TPB) [28] states that behavior is determined by the indirect impact of three factors: attitude, subjective norm, and perceived behavioral control (PBC), mediated through behavioral intention. The TPB has been demonstrated as an efficient theoretical framework for understanding a wide range of consumer behavior, such as pro-environmental behaviors in relation to recycling waste (e.g., [29]), healthy eating behaviors (e.g., [30,31,32,33]), or consumers food purchases (e.g., [34,35,36]). Thus, the classical constructs of the TPB model have been extensively shown to represent consumers’ intentions to engage in specific environmental, eating and purchase behaviors. For instance, the research of Lorenz, Hartmann, and Simons [37] applied the TPB to test the hypothesis that the identification of food with a specific region of origin influences consumer product perception and purchasing intentions. However, these scholars analyzed the benefits associated with the identification of food as an authentic regional product in the case of organic food production. To our knowledge, the current study is the first attempt to apply the TPB model to explain how consumers consider the choice of purchasing authentic regional products after an environmental hazard (*Land of Fires*), which did not truly impact the intrinsic safety of these foods.

Some authors have demonstrated that other relevant psychological constructs are important for explaining food-purchase behavior in the presence of a food or environmental scandal. Considerable attention has been focused on consumer perceptions of risk and trust in public authorities. To the best of our knowledge, there are no studies in the literature that have applied the TPB to explain consumers’ perception of risk and trust surrounding authentic regional food purchases after an environmental scandal. Thus, we have referred to the more general literature on consumer reactions to food scandals.

Risk perception refers to the individual awareness about risky characteristics of a certain behavior [38]. Risk perception can be described as an individual consideration of the probability that an event could happen in the future, is associated with the individual perception of the potential seriousness of its damages [39]. We noted that scholars focused less on the consequences of consumer perceptions surrounding potentially risky food after a high-profile environmental scandal. In this case, risk perception can induce risk-reducing behavior, such as a reduction in the consumption of authentic regional food, which is produced on farmland considered contaminated [40]. We found different studies on consumer cognitions in the literature about microbial food risks, such as salmonella and bird flu [41], whereas few studies analyzed reactions to food risk connected to environmental contaminations, such as dioxins or heavy metals [42,43]. Edgar [44] found that consumers perceive microbial risk as familiar and controllable, while they perceive chemical risks as unfamiliar and uncontrollable. Thus, the lack of scientific considerations on the risk perception of consuming authentic regional food produced in areas with environmental hazards, could be connected to the tendency of consumers of being more familiar with, or knowledgeable about, microbial food risks compared with chemical risks [45], possibly as a consequence of immediate and acute health effects that microbial contaminants could produce [46].

Trust in institutions, which should guarantee environmental and food safety, plays an important role decision-making process during food purchasing [47,48]. Trust influences an individual’s perception of the probability of a risky event [49], and it is related to an individual’s feelings of confidence [50]. If consumers do not have full knowledge to assess risks of a certain food, they may rely on other outlets such as institutions and media [51]. Therefore, they may trust information provided by experts or other sources [52]. For instance, Lobb et al. [41] investigated chicken purchases after the food scandal of bird flu and demonstrated that the addition of trust and risk perception within the TPB allowed for a better understanding of purchasing intentions. In addition, various scientific findings showed that trust influences risk perceptions (e.g., [53,54,55,56]).

As shown above, the TPB constructs plus risk perception and trust were shown to be significant predictors of the intention to purchase specific foods during different food scares (e.g., [41]). Despite that, previous scholars lacked consideration if and in what degree such intentions are translated to actual purchase behaviors. In fact, the translation of behavioral intentions remains the current debate in and the hardest challenge for TPB studies. Different studies on the intention-behavior relationship have revealed that there is a gap between intention and behavior (e.g., [57]). In considering how trust and risk could play an additional and decisive role in predicting intentions to purchase food after an environmental scandal, therefore, it is an important new contribution to verify if consumers’ previous intentions towards purchasing Campania food lead to related purchase behavior.

### 1.2. The Present Study

**Figure 1:**
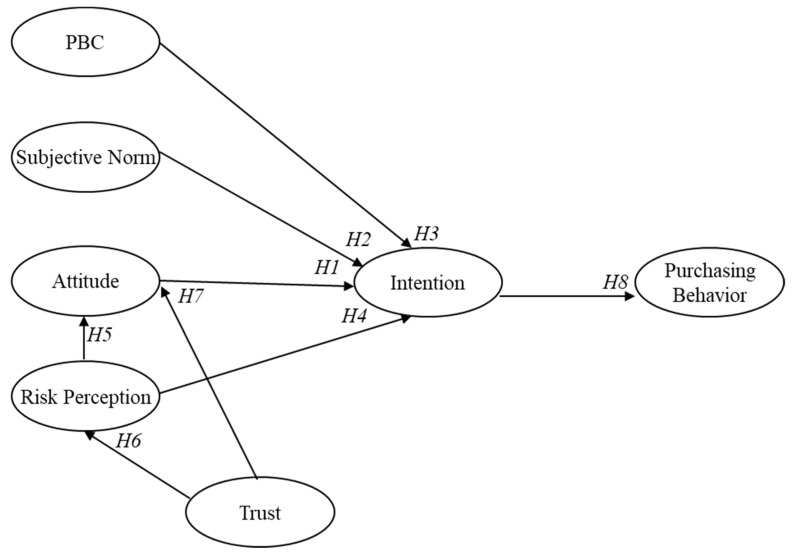
The extended hypothesized model, PBC: perceived behavioral control.

Addressing some of the limitations of previous research, in the present study we proposed a hypothesized extended TPB model, which is shown in Figure 1. Specifically, we tested each of the following hypotheses.

**Hypotheses 1** **(H1).** *Higher consumers’ attitudes related to the purchase of Campania food would predict higher purchasing intentions*.

**Hypotheses 2** **(H2).** *Higher consumers’ subjective norms related to the purchase of Campania food would predict higher purchasing intentions*.

**Hypotheses 3** **(H3).** *Higher consumers’ PBC related to the purchase of Campania food would predict higher purchasing intentions*.

**Hypotheses 4** **(H4).** *Higher consumers’ perception of potential risks of Campania food would predict lower purchasing intentions*.

**Hypotheses 5** **(H5).** *Higher consumers’ perception of potential risks of Campania food would predict lower positive attitudes*.

**Hypotheses 6** **(H6).** *Higher consumers’ trust in institutions would predict lower risk perception*.

**Hypotheses 7** **(H7).** *Higher consumers’ trust in institutions would predict higher positive attitude towards Campania food*.

**Hypotheses 8** **(H8).** *Higher consumers’ purchasing intentions would predict higher subsequent purchases*.

**Hypotheses 9** **(H9).** *Predictors of purchasing intentions (attitude, subjective norm, PBC and risk perception) would have an indirect impact on behavior through intention*.

## 2. Material and Methods

Our study relied on a specifically designed survey carried out in late 2014 on a large sample (1134) of Italian consumers. The survey gathered psychosocial information for a TPB [28] to analyze the role of consumer attitudes, subjective norms and PBC in their intentions and behaviors, including both risk and trust in public institutions, as relevant predictive variables.

### 2.1. Participants and Procedures

Between December 2014 and January 2015, a nationally representative survey was conducted in Italy through a structured questionnaire submitted by GfK, one of the leading market research organization in the world. Consumers who responded to our questionnaire were GfK panelists and the sampling design assures the representativeness of the data according to the following criteria: geographical, size of the city, number of household members, gender, age, education, occupation, and class of income. An additional selection criterion was that respondents had to be responsible for food purchases. This criterion explains the unbalanced number of females and males in the sample. GfK panelists use a specific hardware (an ad hoc customized tablet) for compiling the survey.

A total of 1,134 Italian individuals who are responsible for food purchases within the household (mean age = 53.75, SD = 14.95, ranging from 20 to 89 years old; F = 922; M = 212) have participated to the survey. Most households included 3–4 individuals, and 30% of families had sons between fourteen and eighteen years old. Moreover, the questionnaire included all the items for measuring the traditional TPB constructs, plus trust, risk perception and present (2014) purchase of Campania food, potentially associated with the *Land of Fires* toxic waste scandal.

### 2.2. Measures

The questionnaire included measures of traditional components of TPB (intention, attitude, subjective norm, PBC) in relation to the purchase of Campania food, plus additional variables: risk perception and trust. All measures were measured on 7-point Likert scales. All TPB scales were adapted from [58]. Self-reported purchases of typical Campania products were also measured (Table 1).

Consumer *intentions* were measured using three items. Higher scores indicate a greater intention to purchase Campania food.

*PBC* was measured by three items. Higher scores indicate a greater PBC in relation to purchase Campania food.

Three items with adjective pairs were used to assess consumer *attitudes* towards purchasing Campania food. Higher scores indicate a greater intention to purchase Campania food.

To assess *subjective norm*, three items were used. Higher scores indicate a greater level of subjective norm about purchasing Campania food.

*Trust* in institution was measured by three items adapted from [59]. Higher scores indicate a greater trust in institution in response to the potential risks of Campania food.

Consumers’ risk perception of Campania food was measured by four items adapted from [60]. Higher scores indicate a greater perception of potential risks associated with the consumption of Campania food.

Self-reported consumption of Campania food was assessed with three items.

### 2.3. Data Analyses

TPB was estimated using structural equation modeling (SEM). A hybrid model [61] was developed that simultaneously includes latent variables and a mix of path analysis and confirmatory variables. This approach is a full latent variable model consisting of measurement and structural parameters. The measurement model is related to the within-construct relationship, which regards the relation among measured variables, such as items of a scale, and their respective latent constructs [62]. The structural model concerns with the magnitude and direction of the relations among a series of measured or latent constructs and it is used to verify the hypothesized relationships in the tested model [62]. In the current hybrid model, the measurement model is estimated, and the correlation matrix between constructs and factors serves as an input to estimate the structural coefficients between constructs and latent variables [63]. The adequacy of fit of the SEM models was estimated using a chi-square test and recommended incremental goodness-of-fit indices: the root mean square error of approximation (RMSEA), the comparative fit index (CFI) and the Tucker-Lewis Index (TLI). A nonsignificant chi-square test indicated that the model fits the data well [64]. CFI and TLI cut-off values of at least 0.90 are generally considered to represent an acceptable fit [65,66]. RMSEA value of 0.05 or less indicates a good fit and values up to 0.08 represent errors that approximate those expected in the population [67].

## 3. Results and Discussion

Table 2 shows the descriptive statistics of the items included in the model and the composite reliability of corresponding constructs while Table 3 provides the correlations among constructs and their mean and SD. The items generally showed reasonable variation and were not unduly skewed. The risk measure presented the highest mean, followed by PBC. In general, terms, participants tended to have a negative attitude towards Campania food, a low intention to purchase these foods, and similarly, they reported a low purchasing of these food products. Finally, Italian consumers reported a moderate level of trust in the communications provided by public institutions of the risks related to Campania food. Moreover, consumer intentions to purchase Campania food, attitude and subjective norms showed the strongest correlations with intention and behavior (Table 3).

**Figure 2:**
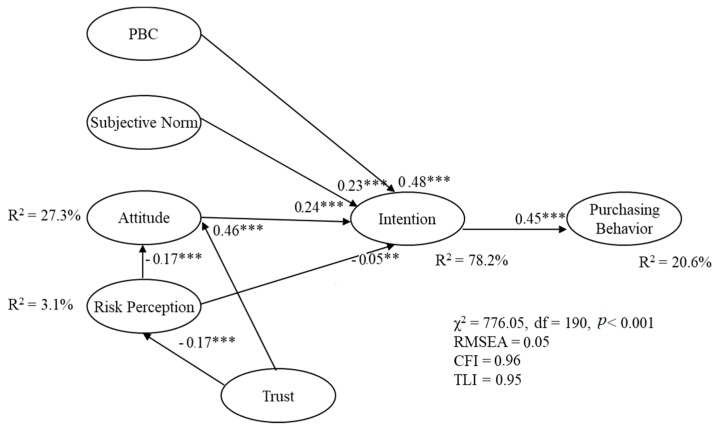
Path model with standardized regression coefficients and standard loadings. Note: *** *p* < 0.001; ** *p* < 0.05.

The goodness-of-fit statistics for the hybrid model were acceptable. The chi-square test was significant (χ^2^ = 776.05, df = 190, *p* < 0.001), and all the other indices pointed to a good fit (RMSEA = 0.05; CFI = 0.96; TLI = 0.95). The parameter estimates were all significant and presented adequate values (from 0.30 to 0.95). No model modification was made, and a conservative strategy of not freeing cross-loadings was followed throughout due to potential impacts on construct validity [68] (Figure 2).

The results confirmed all proposed hypotheses. Intention was positively (*p* < 0.001) determined by attitude (β = 0.24), subjective norm (β = 0.23) and PBC (β = 0.48). These outcomes allowed acceptance of the hypotheses related to the standard TPB model (H1, H2, H3), and demonstrated that the classical TPB constructs, including the motivational cognition of intention, highly predicted Campania food purchasing.

Particularly, attitude, PBC and subjective norm influenced intentions. Such results confirmed the predictive validity of the TPB, extending the important role of its factors in predicting consumer intentions to purchase products after an environmental scandal. Current findings showed that the strongest predictor of consumer behaviors related to the toxic waste scandal was consumers’ PBC. This result suggested that the more Italian consumers could easily control the purchase of Campania food, the more they intended to consume it. From this finding, it could be hypothesized that an important factor for contrasting consumers’ negative reactions after an environmental scandal could be ensuring a higher control on the salubrity of these authentic regional food products, allowing consumers to access certified information surrounding the processes involved in the production of these foods. In fact, the PBC is strictly related to the accessibility of resources that facilitate the control of a behavior, and in this specific case (after a toxic waste scandal), it could be considered the government information as an important external resource that would guarantee a control on the safety of the regional products the consumers are purchasing.

Moreover, H4 was supported by findings related to the negative impact of risk perception on purchasing (β = −0.05; *p* < 0.05). Risk perception predicted attitude (β = −0.17; *p* < 0.001). This outcome supported H5. Trust significantly (*p* < 0.001) predicted attitude (β = 0.46) and risk perception (β = −0.17), confirming H6 and H7. Finally, intention predicted behavior (β = 0.45; *p* < 0.001). Thus, H8 was accepted. Regarding the mediation analyses, PBC (β = 0.21), attitude (β = 0.11), subjective norm (β = 0.10) and risk perception (β = −0.02) indirectly (*p* < 0.001) influenced behavior via intention. These results supported H9. The final model explained 3.1%, 27.3%, 78.2% and 20.6% of the variance of risk perception, attitude, intentions, and behavior, respectively.

The present study revealed the important additional roles of both the risk perception and trust in choosing authentic regional food after an environmental scandal, consistent with other studies, demonstrating their predictive power in other food choices [69]. To illustrate, our hypotheses about the predictive role of the risk perception were in line with the results of a survey in China about the perception of food additive safety and resulting food scares [70] which showed that risk perception was an important factor affecting public food scares and impacted the intention to purchase foods containing additives. Another theoretical support of the impact of risk perception and trust on the intention to consume food considered risky is the research of [59], who investigated Italian consumers’ intention to purchase genetically modified food, considering risk perception, benefit perception, and trust in government institutions. They found that trust in government institutions negatively influenced risk perception and, in turn, negatively affected attitude. Specifically, intention was predicted by PBC, subjective norm, attitude, and risk perception. The current results showed the negative direct impact of risk perception in determining intention to purchase Campania food. This is in line with results obtained by [52]. These authors analyzed the cognitive factors involved in the intention to consume genetically modified food and reported that risk perception, benefit perception, knowledge and trust were factors that affected the attitudes of Taiwanese consumers. Moreover, the current study showed that risk perception also has a negative indirect impact on intentions mediated by attitude. This mediation role of attitude was also identified by Stefani and colleagues [71] considered the impact of risk on attitude, analyzing how risk was, in turn, predicted by different types of trust (trust in media, trust in chain, trust in policy, etc.). Their results showed that trust in chains and in other media influenced risk perception, which in turn showed a negative effect on attitude. The current study obtained evidence in line with previous studies [52,59,72], corroborating the idea that risk perception is directly affected by trust. Moreover, in line with the findings of [73] which examined consumer attitudes towards genetically engineered salmon, attitude was more predicted by trust than risks: trust positively affected attitude, while risk negatively affected attitude. Thus, trust was identified as a crucial element in reducing the level of risk perception in Italy.

## 4. Conclusions

This study investigated the psychosocial factors involved in the reduction in purchasing authentic regional food products after the *Land of Fires* scandal, and these findings pave the way for future studies that aim to both investigating other factors to explain this phenomenon and replicating this research in other contexts, such as regions with contaminated soil.

The most important result, whit theoretical and empirical implications, is that the influence of trust on the risk perception may offer a relevant potential contribution to the future development of effective safety policy communication. Until now, Italian policy has focused only on an attempt to reduce the risk perception surrounding Campania food, providing information that supports its safety. As in the case of GM foods [74], after the environmental scandal Italian consumers still perceive authentic regional Campania food products as having some risk, even if the authorities did not find any connection between the environmental hazard in this region and its food safety. This aspect strongly highlights the lack of trust in institutional information and thus their inability to reassure consumers and the lack of responsible engagement in territorial communities [75].

Our results suggest that the traditional contents government communication (such as details about the waste scandal, responsibilities, and long-term perspective about pollution area) may not be useful to change people’s mind about produces from the “Land of Fire”. On the contrary, mass media’s communication, based on threatening and frightening topics such as indexes about death, cancer, and malformations in Campania area [76], appeals to fear and has heightened the risk perception on food produces from Campania. Recent studies [77,78], instead, demonstrate that low-fear appealing messages tend to evoke more fearful emotions and more intentions to engage in pro-environmental behavior, rather than the high-fear appealing messages. State communication should rely on appropriate low-fear messages that can increase people’s responsiveness to environmental appeals and change their behavioral intentions.

Since the present study revealed the importance of resource accessibility to facilitate the control of consumer purchase behavior (PBC), if the government provides information to reduce the risk perception and increase consumers’ trust, they should be able to ensure the safety of the regional products that consumers are purchasing. Once again, labeling foods and process attributes as safe could help mitigate consumer concerns.

One limitation of this study is that the only consideration of trust was towards three different institutional interventions. Including other types of trust, such as trust in mass media, could further clarify its role in risk perception. Moreover, notable differences in trust perception could emerge, considering the different actors involved in the food chain. To avoid overestimating the importance of risk perception and trust, which are certainly not the only factors that can contribute within the TPB to the formation of food purchasing behavior, further studies may also include the individual cognitive evaluation of the probability that risky events could occur. Moreover, another important dimension that could help scholars to deepen the perception of risk in Italian individuals towards the regional food, which are produced in areas affected by the *Land of Fires* toxic waste scandal, may be their attachment to the Italian farmland and the Italian food culture [79]. The distorted nature of the assessment of risk may also be incurred by optimistic or pessimistic subjective perspective. Therefore, in terms of food safety, future research may consider optimism and pessimism (e.g., [80]) as two distinct dimensions of consumers’ trust. Future research should study the ways in which social cognitive models can be extended to improve their predictive function. There are many additional variables that could be included into the TPB framework (see [30]), such as the health locus of control [81]. The predictive capacity of the health locus of control has already been tested in previous studies [82] and should be evaluated in response to this specific behavior as it is not generalizable across behaviors but must be assessed in a situation-specific manner. Finally, the data gathered by this study might contribute to a fine-grain model of TPB based on a multi agent system approach [83] complemented with bio-inspired cognitive modeling [84]. Such a model, able to simulate different scenarios, could represent a useful tool for policy makers.

## Figures and Tables

**Table 1:** Items included in the model.

Items
***Intention***
I.1 I intent to purchase Campania food
I.2 I plan to purchase Campania food
I.3 I want to purchase Campania food
***Perceived behavioral control***
PBC.1 Controlling my purchase of Campania food is easy
PBC.2 Whether or not I purchase Campania food is completely up to me
PBC.3 During my purchases, if I want, I can be sure of the healthiness of the Campania products
***Attitudes***
A.1 Purchasing of Campania food is worthless/valuable
A.2 Purchasing of Campania food is positive/negative
A.3 Purchasing of Campania food is interesting/boring
***Subjective norm***
SN.1 People who are important to me think I can purchase Campania food
SN.2 People who are important to me would approve my purchasing of Campania food
SN.3 People who are important do not want me to purchase Campania food (R)
***Trust in the institutions***
T.1 Do you trust the Italian Government initiatives (certification system and traceability) in response to the potential risks of Campania food?
T.2 Do you trust the public research initiatives (University and Public research system) in response to the potential risks of Campania food?
T.3 Do you trust the Campania farmers’ initiatives in response to the potential risks of Campania food?
***Risk Perception about Campania food***
R.1 Do you think that there are potential risks associated with the consumption of Campania food?
R.2 Do you think that potential risks associated with the consumption of Campania food are known to the scientific community?
R.3 Do you think that potential risks associated with the consumption of Campania food are acceptable?
R.4 How do you think that Campania food is risky for the community?
***Campania food consumption***
B.1 How often do you purchase mozzarella produced in Campania
B.2 How often do you purchase fruit and vegetable produced in Campania
B.3 How often do you purchase other Campania products?

**Table 2:** Means and standard deviations of each items.

Items	M	SD	CR
***Intention***			
I.1	2.66	1.93	0.62
I.2	2.51	1.88	
I.3	2.62	1.89	
***Perceived behavioral control***			
PBC.1	3.18	1.97	0.58
PBC.2	4.58	2.17	
PBC.3	4.11	2.19	
***Attitude***			0.69
A.1	2.79	1.62	
A.2	2.80	1.63	
A.3	2.82	1.67	
***Subjective norm***			
SN.1	4.07	2.20	0.60
SN.2	3.30	1.90	
SN.3	3.07	1.90	
***Trust in the institutions***			
T.1	3.91	1.87	0.65
T.2	3.74	1.74	
T.3	3.55	1.84	
***Risk Perception about Campania food***			
R.1	4.51	1.96	0.71
R.2	5.07	1.94	
R.3	2.54	1.97	
R.4	5.67	1.72	
***Campania food consumption***			
B.1	2.04	1.04	0.62
B.2	2.67	1.67	
B.3	2.72	1.60	

Note: M = Mean, SD = Standard Deviation, CR = Composite Reliability.

**Table 3:** Descriptive finding and correlations between study variables.

	1.	2.	3.	4.	5.	6.	7.	M	SD
1. Intention	1							2.60	1.81
2. PBC	0.56 **	1						3.96	1.59
3. Attitude	0.74 **	0.51 **	1					2.80	1.56
4. Subjective norm	0.62 **	0.43 **	0.65 **	1				3.50	1.50
5. Trust	0.37 **	0.38 **	0.41 **	0.33 **	1			3.75	1.58
6. Risk Perception	0.03	0.10 **	−0.02	−0.01	0.05	1		4.44	1.30
7. Purchasing Behavior	0.27 **	0.07 *	0.21 **	0.22 **	0.08 **	−0.01	1	2.47	1.18

Note: ** *p* < 0.001; * *p* < 0.05.

## References

[1] Van der Linden S. (2015). The social-psychological determinants of climate change risk perceptions: Towards a comprehensive model. J. Environ. Psychol..

[2] Liu S., Chiang Y.T., Tseng C.C., Ng E., Yeh G.L., Fang W.T. (2018). The Theory of Planned Behavior to Predict Protective Behavioral Intentions against PM2.5 in Parents of Young Children from Urban and Rural Beijing, Chin. Int. J. Environ. Res. Public Health.

[3] Rieger J., Kuhlgatz C., Anders S. (2016). Food scandals, media attention and habit persistence among desensitised meat consumers. Food Policy.

[4] Cicia G., Caracciolo F., Cembalo L., Del Giudice T., Grunert K.G., Krystallis A., Lombardi P., Zhou Y. (2016). Food safety concerns in urban China: Consumer preferences for pig process attributes. Food Control.

[5] Masters W.A., Hall A., Martinez E.M., Shi P., Singh G., Webb P., Mozaffarian D. (2016). The nutrition transition and agricultural transformation: A Preston curve approach. Agric. Econ..

[6] Brunori G., Malandrin V., Rossi A. (2013). Trade-off or convergence? The role of food security in the evolution of food discourse in Italy. J. Rural Stud..

[7] Mazzocchi M., Stefani G., Henson S.J. (2004). Consumer welfare and the loss induced by withholding information: The case of BSE in Italy. J. Agric. Econ..

[8] Mazzocchi M., Lobb A., Bruce Traill W., Cavicchi A. (2008). Food scares and trust. A European study. J. Agric. Econ..

[9] Misso R. (2009). Consumer health protection and Information and Communication Technology. Proceedings of the 113th Seminar, Chania, Crete, Greece, 3–6 September 2009 (No. 58094).

[10] Borrello S., Brambilla G., Candela L., Diletti G., Gallo P., Iacovella N., Iovane G., Limone A., Migliorati G., Pinto O. (2008). Management of the 2008 “buffalo milk crisis” in the Campania Region under the perspective of consumer protection. Organohal. Compd..

[11] Giuliano G. (1995). Ground water in the Po basin: Some problems relating to its use and protection. Sci. Total Environ..

[12] Khan S., Cao Q., Zheng Y.M., Huang Y.Z., Zhu Y.G. (2008). Health risks of heavy metals in contaminated soils and food crops irrigated with wastewater in Beijing, China. Environ. Pollut..

[13] Zhang J., Mauzerall D.L., Zhu T., Liang S., Ezzati M., Remais J.V. (2010). Environmental health in China: Progress towards clean air and safe water. Lancet.

[14] Wilcock A., Pun M., Khanona J., Aung M. (2004). Consumer attitudes, knowledge and behaviour: A review of food safety issues. Trends Food Sci. Technol..

[15] McMichael A.J. (2001). Impact of climatic and other environmental changes on food production and population health in the coming decades. Proc. Nutr. Soc..

[16] Adda J. (2007). Behavior towards health risks: An empirical study using the “Mad Cow” crisis as an experiment. J. Risk Uncertain.

[17] Ding Y., Veeman M.M., Adamowicz W.L. (2013). The influence of trust on consumer behavior: An application to recurring food risks in Canada. J. Econ. Behav. Organ..

[18] Piggott N.E., Marsh T.L. (2004). Does food safety information impact U.S. meat demand?. Am. J. Agric. Econ..

[19] Verbeke W., Ward R.W. (2001). A fresh meat almost ideal demand system incorporating negative TV press and advertising impact. Agric. Econ..

[20] Vringer K., van der Heijden E., van Soest D., Vollebergh H., Dietz F. (2017). Sustainable consumption dilemmas. Sustainability.

[21] Bertolotti M., Catellani P. (2015). Agreement with climate change policies: Framing the future and national versus supra-national identity. Eur. J. Soc. Psychol..

[22] Meyer S.B., Wilson A.M., Calnan M., Henderson J., Coveney J., McCullum D., Pearce A.R., Ward P., Webb T. (2017). In the interest of food safety: A qualitative study investigating communication and trust between food regulators and food industry in the UK, Australia and New Zealand. BMC Public Health.

[23] Rieger J., Weible D., Anders S. (2017). “Why some consumers don’t care”: Heterogeneity in household responses to a food scandal. Appetite.

[24] Lu Y., Song S., Wang R., Liu Z., Meng J., Sweetman A.J., Jenkins A., Ferrier R.C., Li H., Luo W. (2015). Impacts of soil and water pollution on food safety and health risks in China. Environ. Int..

[25] Strauss B. (2009). Spartacus War.

[26] Senior K., Mazza A. (2004). Italian “Triangle of death” linked to waste crisis. Lancet Oncol..

[27] Flora A. (2015). La Terra dei Fuochi: Ambiente e politica industriale nel Mezzogiorno. Rivista Economica del Mezzogiorno.

[28] Ajzen I. (1991). The theory of planned behaviour. Organ. Behav. Hum. Decis. Process..

[29] Carfora V., Caso D., Sparks P., Conner M. (2017). Moderating effects of pro-environmental self-identity on pro-environmental intentions and behaviour: A multi-behaviour study. J. Environ. Psychol..

[30] Armitage C.J., Conner M. (2001). Efficacy of the theory of planned behaviour: A meta-analytic review. Br. J. Soc. Psychol..

[31] Canova L., Manganelli A.M. (2016). Fruit and vegetables consumption as snacks among young people. The role of descriptive norm and habit in the theory of planned behavior. Test. Psychometr. Methodol. Appl. Psychol..

[32] Carfora V., Caso D., Conner M. (2017). Randomised controlled trial of a text messaging intervention for reducing processed meat consumption: The mediating roles of anticipated regret and intention. Appetite.

[33] Carfora V., Caso D., Palumbo F., Conner M. (2018). Promoting water intake. The persuasiveness of a messaging intervention based on anticipated negative affective reactions and self-monitoring. Appetite.

[34] Mari S., Tozzo B., Capozza D., Ravarotto L. (2012). Are you cooking your meat enough? The efficacy of the Theory of Planned Behaviour in predicting a best practice to prevent salmonellosis. Food Res. Int..

[35] Lombardi A., Carfora V., Cicia G., Del Giudice T., Lombardi P., Panico T. (2017). Exploring willingness to pay for QR code labeled extra-virgin olive oil: An application of the theory of planned behavior. Int. J. Food Syst. Dyn..

[36] Wong S.L., Hsu C.C., Chen H.S. (2018). To Buy or Not to Buy? Consumer Attitudes and Purchase Intentions for Suboptimal Food. Int. J. Environ. Res. Public Health.

[37] Lorenz B.A., Hartmann M., Simons J. (2015). Impacts from region-of-origin labeling on consumer product perception and purchasing intention—Causal relationships in a TPB based model. Food Qual. Prefer..

[38] Donizzetti A.R. (2009). Risk perception in adolescence: Construction and validation of new scales | [La percezione del rischio in adolescenza: Costruzione e validazione di strumenti di rilevazione]. Psicologia della Salute.

[39] Homburg A., Stolberg A. (2006). Explaining pro-environmental behavior with a cognitive theory of stress. J. Environ. Psychol..

[40] Yeung R.M.W., Yee W.M.S. (2003). Risk reduction: An insight from the UK poultry industry. Nutr. Food Sci..

[41] Lobb A.E., Mazzocchi M., Traill W.B. (2007). Modelling risk perception and trust in food safety information within the theory of planned behaviour. Food Qual. Prefer..

[42] Burger J. (1998). Gender differences in attitudes about fish safety in a coastal population. J. Toxicol. Environ. Health Part A.

[43] Kher S.V., De Jonge J., Wentholt M.T.A., Deliza R., Cunha de Andrade J., Cnossen H.J., Luijckx N.B.L., Frewer L.J. (2013). Consumer perceptions of risks of chemical and microbiological contaminants associated with food chains: A cross-national study. Int. J. Consum. Stud..

[44] Edgar J. (2004). Future impact of food safety issues on animal production and trade: Implications for research. Aust. J. Exp. Agric..

[45] Fife-Schaw C., Rowe G. (1996). Public perceptions of everyday food hazards: A psychometric study. Risk Anal..

[46] Starbird S.A., Walker G.A. Determinants of consumer perceptions of food safety risk. Proceedings of the 14th Annual Conference of the International Food and Agribusiness Management Association.

[47] Glaeser E.L., Laibson D.I., Scheinkman J.A., Soutter C.L. (2000). Measuring trust. Q. J. Econom..

[48] Hobbs J.E., Goddard E. (2015). Consumers and trust. Food Policy.

[49] Bonoma T.V., Johnston W.J. (1979). Locus of control, trust, and decision making. Decis. Sci..

[50] Luhmann N. (1979). Trust and Power.

[51] Ding Y., Veeman M.M., Adamowicz W.L. (2015). Functional food choices: Impacts of trust and health control beliefs on Canadian consumers’ choices of canola oil. Food Policy.

[52] Chen M.F., Li H.L. (2007). The consumer’s attitude toward genetically modified foods in Taiwan. Food Qual. Prefer..

[53] Frewer L.J., Howard C., Hedderley D., Shepherd R. (1997). Consumer attitudes towards different food-processing technologies used in cheese production: The influence of consumer benefit. Food Qual. Prefer..

[54] Frewer L.J. (1999). Risk perception, social trust, and public participation in strategic decision making: Implication for emerging technologies. Ambio.

[55] Trumbo C.W., McComas K.A. (2003). The function of credibility in information processing for risk perception. Risk Anal..

[56] Viklund M.J. (2003). Trust and Risk Perception in Western Europe: A Cross-National Study. Risk Anal..

[57] Sheeran P. (2002). Intention-behaviour relations: A conceptual and empirical overview. Eur. Rev. Soc. Psychol..

[58] Ajzen I., Sheikh S. (2013). Action versus inaction: Anticipated affect in the theory of planned behaviour. J. Appl. Soc. Psychol..

[59] Prati G., Pietrantoni L., Zani B. (2012). The prediction of intention to consume genetically modified food. Test of an integrated psychosocial model. Food Qual. Prefer..

[60] Albanesi C., Prati G., Pietrantoni L., Zani B., Cicognani E., Prati G., Zani B. (2011). La percezione del rischio da uranio impoverito nella popolazione. Uranio Impoverito.

[61] Hancock G.R., Samuelsen K.M. (2007). Advances in Latent Variable Mixture Models.

[62] Stephenson M.T., Holbert R.L. (2003). A Monte Carlo simulation of observable versus latent variable structural equation modeling techniques. Commun. Res..

[63] Kline R. (1998). Principles and Practice of Structural Equation Modeling.

[64] Iacobucci D. (2010). Structural equations modeling: Fit Indices, sample size, and advanced topics. J. Consum. Psychol..

[65] Tucker L.R., Lewis C. (1973). A reliability coefficient for maximum likelihood factor analysis. Psychometria.

[66] Bentler P.M. (1990). Comparative fit indexes in structural models. Psychol. Bull..

[67] Browne M.W., Cudeck R. (1992). Alternative ways of assessing model fit. Sociol. Methods Res..

[68] Hair J.F., Black W.C., Babin B.J., Anderson R.E., Tatham R.L. (2006). Multivariate Data Analysis.

[69] Conner M., Kirk S.F.L., Cade J.E., Barrett J.H. (2001). Why do women use dietary supplements? The use of the theory of planned behaviour to explore beliefs about their use. Soc. Sci. Med..

[70] Wu L., Zhong Y., Shan L., Qin W. (2013). Public risk perception of food additives and food scares. The case in Suzhou, China. Appetite.

[71] Stefani G., Cavicchi A., Romano D., Lobb A.E. (2008). Determinants of intention to purchase chicken in Italy: The role of consumer risk perception and trust in different information sources. Agribusiness.

[72] Siegrist M. (1999). A causal model explaining the perception and acceptance of gene technology. J. Appl. Soc. Psychol..

[73] Qin W., Brown J.L. (2008). Factors explaining male/female differences in attitudes and purchase intention toward genetically engineered salmon. J. Consum. Behav..

[74] Frewer L.J., Scholderer J., Bredahl L. (2003). Communicating about the risks and benefits of genetically modified foods: The mediating role of trust. Risk Anal..

[75] Natale A., Di Martino S., Procentese F., Arcidiacono C. (2016). De-growth and critical community psychology: Contributions towards individual and social well-being. Futures.

[76] Sacfuto F., La Barbera F. (2016). Protest AgainstWaste Contamination in the ‘Land of Fires’: Psychological Antecedents for Activists and Non-activists. J. Community Appl. Soc. Psychol..

[77] Chen M.F. (2016). Impact of fear appeals on pro-environmental behavior and crucial determinants. Int. J. Advert..

[78] Hartman P., Apaloaza V., D’Souza C., Barrutia J.M., Echebarria C. (2014). Environmental threat appeals in green advertising: The role of fear arousal and coping efficacy. Int. J. Advert..

[79] Bonaiuto M., Alves S., De Dominicis S., Petruccelli I. (2016). Place attachment and natural hazard risk: Research review and agenda. J. Environ. Psychol..

[80] Sarabia-Sanchez F., Rodriguez-Sanchez C. (2016). The role of credibility and negative feelings in comparative perceptual bias related to environmental hazards. J. Environ. Psychol..

[81] Donizzetti A.R., Petrillo G. (2015). Validazione della versione per adulti della Health Locus of Control Scale (HLCS) [Health Locus of Control Scale for adults: A validation study]. Psicologia della Salute.

[82] Armitage C.J., Norman P., Conner M. (2002). Can the Theory of Planned Behaviour mediate the effects of age, gender and multidimensional health locus of control?. Br. J. Health Psychol..

[83] Scalco A., Ceschi A., Sartori R. (2018). Application of Psychological Theories in Agent-Based Modeling: The Case of the Theory of Planned Behavior. Nonlinear Dyn. Psychol. Life Sci..

[84] Pacella D., Ponticorvo M., Gigliotta O., Miglino O. (2017). Basic emotions and adaptation. A computational and evolutionary model. PLoS ONE.

